# The Proteome of Bone Marrow Multipotent Mesenchymal Stromal Cells Undergoes Significant Alterations in Acute Leukemia Patients at the Onset and During Treatment

**DOI:** 10.3390/ijms27167402

**Published:** 2026-08-19

**Authors:** Nataliya A. Petinati, Aleksandra V. Sadovskaya, Irina N. Shipounova, Nina I. Drize, Anastasia N. Vasilyeva, Olga A. Aleshina, Alexandra S. Paderina, Olga S. Pokrovskaya, Larisa A. Kuzmina, Igor P. Smirnov, Olga V. Pobeguts, Georgij P. Arapidi, Maria A. Lagarkova, Elena N. Parovichnikova

**Affiliations:** 1 National Medical Research Center for Hematology, Moscow 125167, Russiashipunova.i@blood.ru (I.N.S.);; 2 Department of Immunology, Faculty of Biology, Federal State Budget Educational Institution of Higher Education M.V., Lomonosov Moscow State University, Moscow 119991, Russia; 3 Lopukhin Federal Research and Clinical Center of Physical-Chemical Medicine of Federal Medical Biological Agency, Moscow 119435, Russia; 4 Shemyakin-Ovchinnikov Institute of Bioorganic Chemistry of the Russian Academy of Sciences, Miklukho-Maklaya 16/10, Moscow 117997, Russia; 5 Moscow Center for Advanced Studies, 20, Kulakova Str., Moscow 123592, Russia

**Keywords:** multipotent mesenchymal stromal cells, acute leukemia, allo-HSCT, proteome, metabolism, bone marrow niche, ECM remodeling, mitochondrial dysfunction

## Abstract

The bone marrow stromal microenvironment is damaged in patients with acute leukemia. The aim of this study was to analyze changes associated with the extracellular matrix, mitochondrial function, and vesicular transport in the proteome of multipotent mesenchymal stromal cells (MSCs) in patients at the onset, in remission, before, and 1–3 months after allogeneic hematopoietic stem cell transplantation (allo-HSCT). The study included paired MSCs samples from the bone marrow of 12 patients at the onset and in remission of acute leukemia (4 ALL, 8 AML) and eight patients before and after allo-HSCT (4 ALL, 4 AML). MSCs from eight healthy donors were used as a control. The growth characteristics and the proteome subsets describing extracellular matrix, mitochondria, and vesicular formation were studied. The proteome of the patients’ MSCs differed significantly from that of the donor MSCs, both at the onset and in remission. Changes noted in the composition of extracellular matrix proteins may affect cell adhesion and access to growth factors. Significant changes were revealed in proteins affecting mitochondrial function. Vesicular transport proteins also differed between the donor and patient groups. Unexpectedly, no differences were found between the MSCs of donors and patients before and after allo-HSCT.

## 1. Introduction

The fate of hematopoietic stem cells (HSCs) in the bone marrow depends heavily on their microenvironment. Mesenchymal stem cells and their descendants, including multipotent mesenchymal stromal cells (MSCs), are an essential part of this microenvironment and regulate HSCs through intercellular contact, soluble factors, and niche formation. These interactions are pivotal for proliferation, differentiation, and maintenance of the HSC pool [1,2]. The extracellular matrix (ECM), a crucial part of the bone marrow niche, is a complex three-dimensional network of macromolecules that performs key regulatory and structural functions. Mesenchymal cells are the main contributors to ECM secretion. Both stromal and hematopoietic stem and progenitor cells require ECM signaling for survival, proliferation, migration, and differentiation [3,4]. ECM stiffness and composition contribute to the differentiation choice of MSCs into bone, cartilage, or adipose tissue [5,6]. ECM serves as a reservoir for growth factors and cytokines, controlling their availability to cells and regulating the maintenance of the hematopoietic stem cell pool. In acute leukemia, ECM undergoes critical changes that are permissive for tumorigenesis and prohibitive for normal hematopoiesis [7].

Acute leukemia also induces the transformation of the hematopoietic niche cells, including MSCs. It has been shown that in patients with acute leukemia, the properties of MSCs and their ability to maintain normal hematopoiesis change [8,9]. The increasing need to support leukemic growth alters MSC metabolism [10]. A vital part of cellular metabolism is respiration and the balance between glycolysis and oxidative phosphorylation, which produces reactive oxygen species (ROS). This applies to both MSCs and HSCs. Normally, quiescent HSCs maintain low concentrations of ROS, which prevents their transition to proliferation and differentiation, preserving their pool [11,12]. Regenerative stress after allogeneic hematopoietic stem cell transplantation (allo-HSCT) leads to the activation of mitochondrial respiration and the accumulation of ROS, which, in the absence of protective signals from the stromal microenvironment, can lead to depletion of the HSC pool and hematopoietic failure [12]. ROS upregulation impairs the clonogenicity and differentiation of MSCs, as well as their ability to support HSCs [13].

Metabolism, the expression of surface molecules, the differentiation capacity, the secretion of cytokines, and the extracellular matrix are important for the characterization of MSCs. It is known that many of the cytokines and growth factors produced by MSCs are secreted within exosomes and vesicles [14].

The treatment administered to patients also affects stromal cell function. For example, alterations in MSCs’ adipose differentiation and aging were detected both at the onset of the disease and during treatment [15,16]. Drugs damage not only hematopoietic progenitors but also other cells, including MSCs, and, thus, can have a detrimental effect on the stromal microenvironment. Doxorubicin has been shown to induce blockage of MSC differentiation and reduced niche capacity to maintain HSCs [17]. Cytarabine, daunorubicin, and vincristine altered the phenotype, differentiation capacity, and gene expression of MSCs [18]. Therefore, it is important to investigate the dynamics of MSC changes during treatment. Allo-HSCT is a complex therapeutic approach that affects the bone marrow microenvironment by high-dose chemotherapy conditioning, the elimination of native recipient hematopoietic cells, and niche repopulation by donor cells.

We have previously studied growth parameters, surface phenotype, gene expression profiles, and the hematopoiesis supporting the ability of MSCs from the bone marrow of acute leukemia patients at various time points [9,19,20,21]. Proteome analysis was the next step in our research. We collected two groups of paired samples. One included MSCs from the same patients at diagnostic and in remission time points; another included MSCs from patients before and after allo-HSCT. The challenge of receiving two MSC samples from the same patient resulted in the small sample number and the variety of the time-points.

The aim of this work was to analyze the change in the MSC proteome of patients with acute leukemia over time—at the onset of the disease, in remission, and after allo-HSCT.

## 2. Results

Proteomic analysis was performed on cells after passage 3 (P3). The characteristics of the cultures that were analyzed are shown in Table 1.

Two sets of paired patient samples are presented: one group was analyzed at the onset and in remission of acute leukemia; another was analyzed before and after allo-HSCT. Groups in remission and before allo-HSCT are clinically very similar and display similar cultural characteristics. As shown previously, the culture characteristics of patients’ cells differ from donors’ cells at all sampling points [19,22].

While it was our initial intention to analyze AML and ALL groups separately, it was difficult to obtain MSC samples of the same patient at two required time points due to the poor growth capabilities of AL-MSCs and clinical limitations (such as treatment resistance, graft failure, refusal of treatment, etc.). This has resulted in a small sample size, even when put together, as pointed out above; separate analysis was considered unfeasible.

### 2.1. General Proteome Analysis

**Figure 1:**
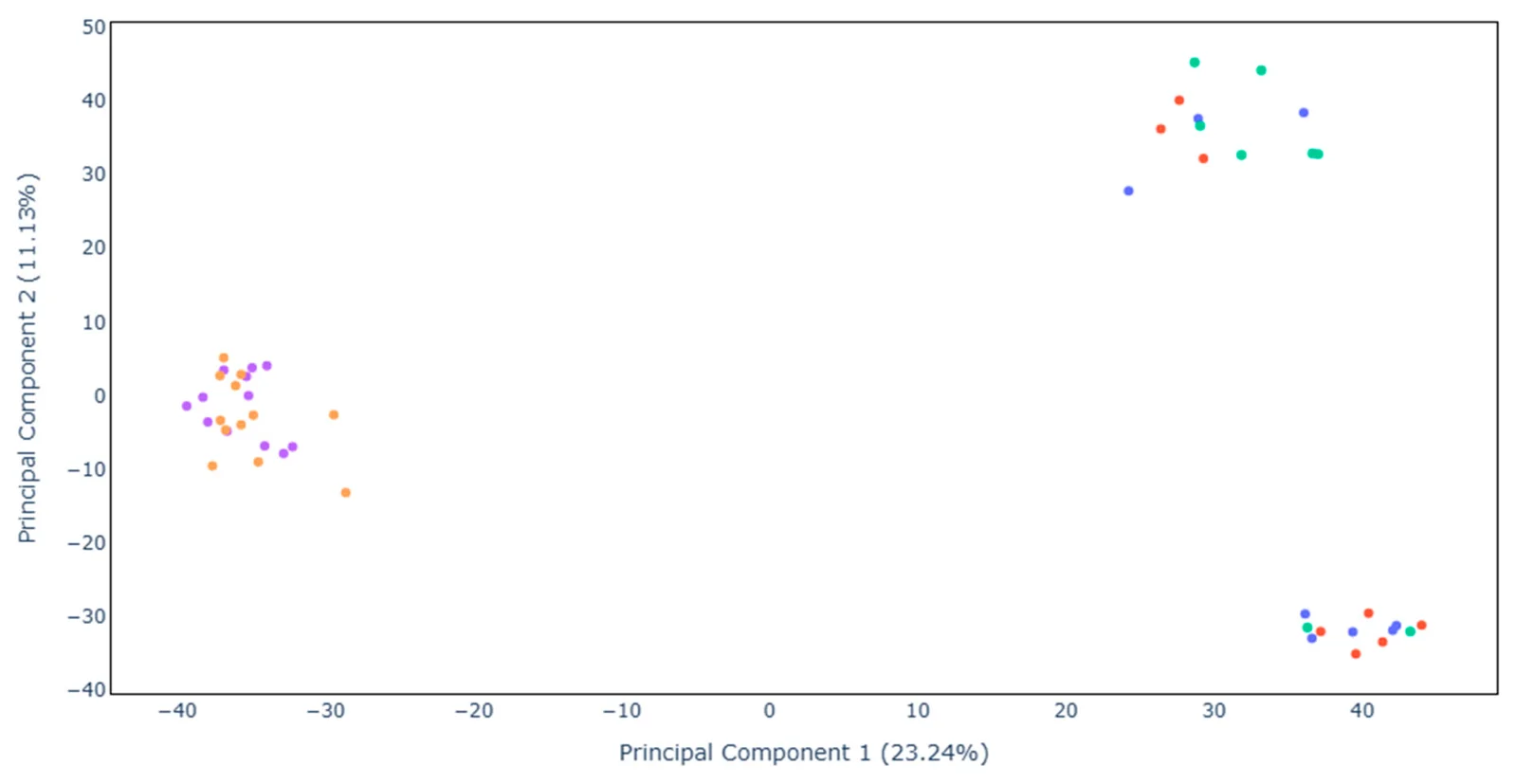
Principal component analysis showing clusterization of the samples. Green dots represent samples of the donor group; purple dots represent samples of the onset group; orange dots represent samples of the remission group; blue dots represent samples of the group before allo-HSCT; red dots represent samples of the group after allo-HSCT.

In total, 6117 proteins were identified across 48 samples. Principal component analysis (PCA) showed clustering of the samples (Figure 1). PC1 explains 23.24% of the variability and PC2 explains 11.13%. Together, they explain more than 37% of the total variance.

PCA clustered samples from acute leukemia patients at the onset and in remission, separately from donors’ and allo-HSCT patients’ ones.

The consequent comparative analysis of proteomes revealed no significant differences in protein composition between MSCs from donors and patients before and after allo-HSCT.

In the MSCs of the patients at the onset of acute leukemia, 98 proteins were significantly (adjusted *p* < 0.05, fold change > 2) lower than in the donor MSCs and 23 were higher (Supplementary Table S1). In remission, 88 proteins were downregulated compared to donor cells and 42 were upregulated (Supplementary Table S2). Ninety-five proteins were changed similarly at the onset and in remission, compared to donors’ cells. PICALM, NAP1L1, PPP1CB, SLC25A6, SERPINB6, TGFBI, TRIOBP, PSMC3, FUBP3, YWHAH, RPL23, RPS9, G3BP2, ARF4, ERO1A, KHSRP, LGALS1, NDUFS5, EXOC6B, ZRANB2, SGSH, CTBP2, PSMB9, SP100, and SSRP1 differed significantly, exclusively between the MSCs of the patients at the onset and donors. OSTC, ARMCX3, MYO1B, GNAS, PLIN3, NNMT, GLUD1, IMMT, ST13, RPS10, CKAP5, UBR4, PGM3, UACA, PMPCA, NSFL1C, MRPS31, UNC45A, MYO9B, NID2, TEX10, IPO4, CPD, LPCAT2, DHX29, MMS19, DDX54, TBC1D10A, SENP3, CIAO1, PDPR, UBASH3B, TXNRD2, HEATR5A, and PSEN1 were expressed differently only in remission when compared with donors.

**Figure 2:**
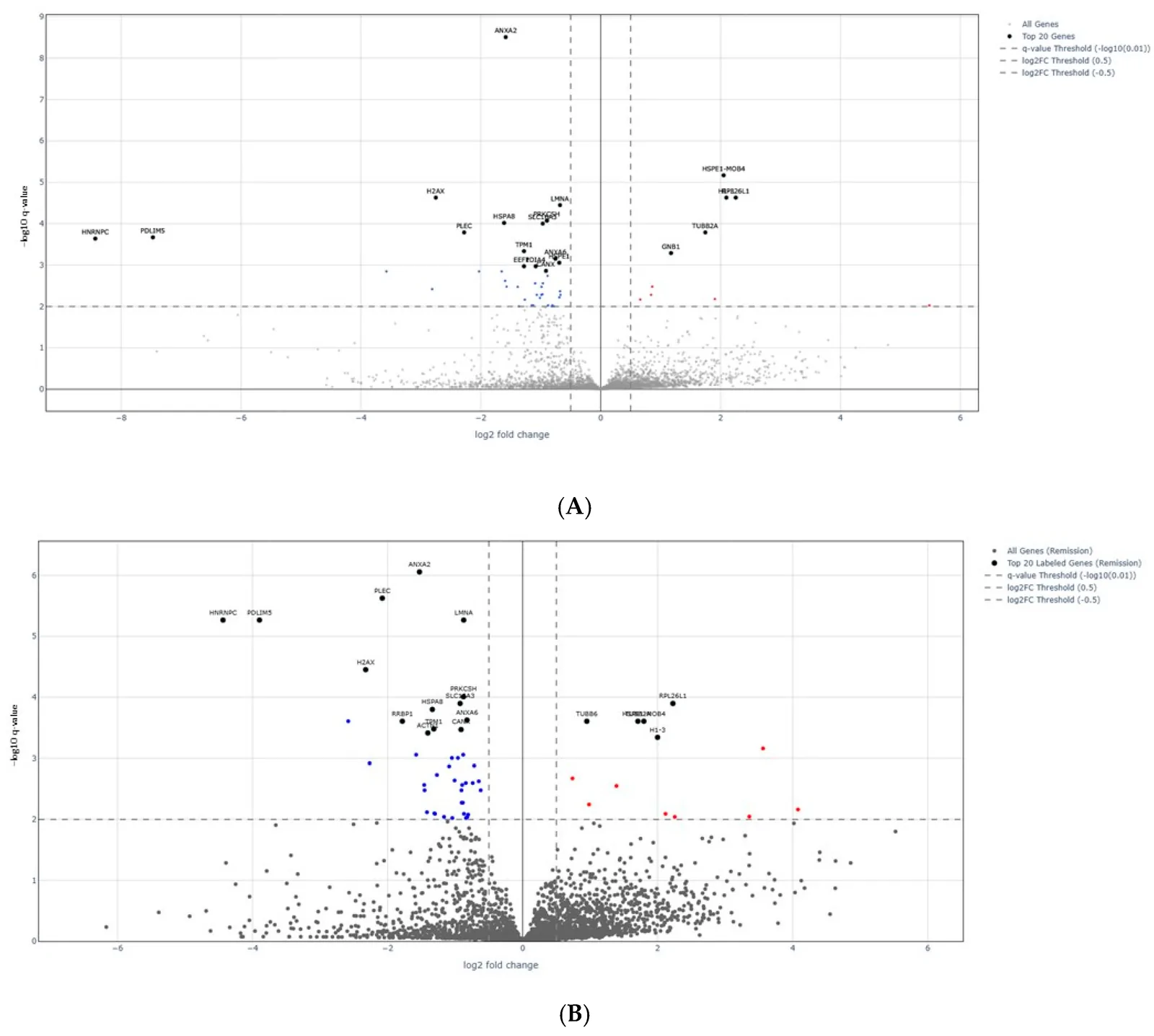
Volcano plots showing significantly (adjusted *p* < 0.05, fold change > 2) differing proteins between groups. The top 20 proteins are labeled: (**A**) proteins significantly upregulated (red) and downregulated (blue) in the MSCs of patients at the onset of acute leukemia compared to donor MSCs; and (**B**) proteins significantly upregulated (red) and downregulated (blue) in MSCs of the patients at the remission of acute leukemia compared to donor MSCs.

Pairwise comparisons between donor and patient cells are presented as volcano plots in Figure 2.

**Figure 3:**
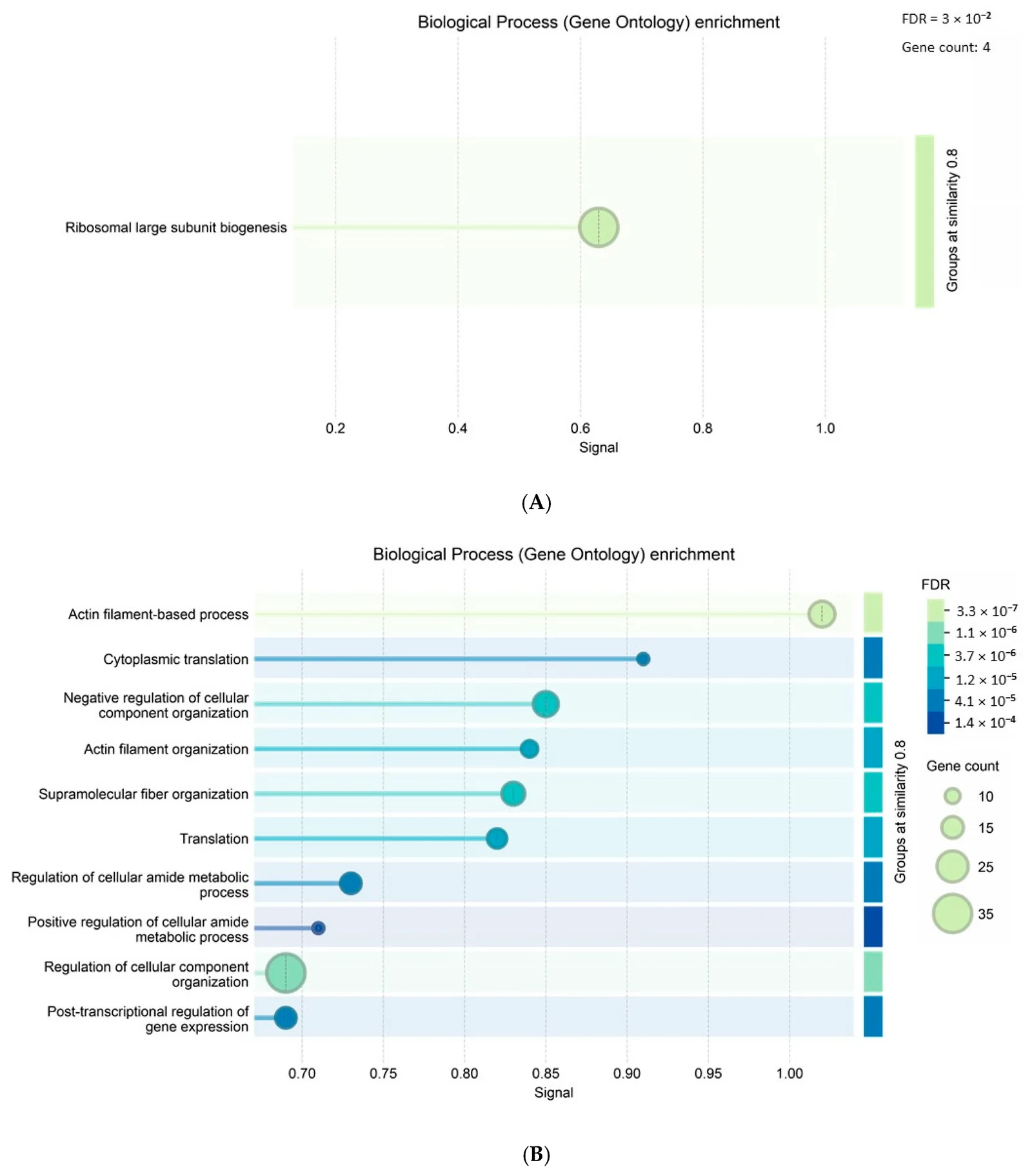

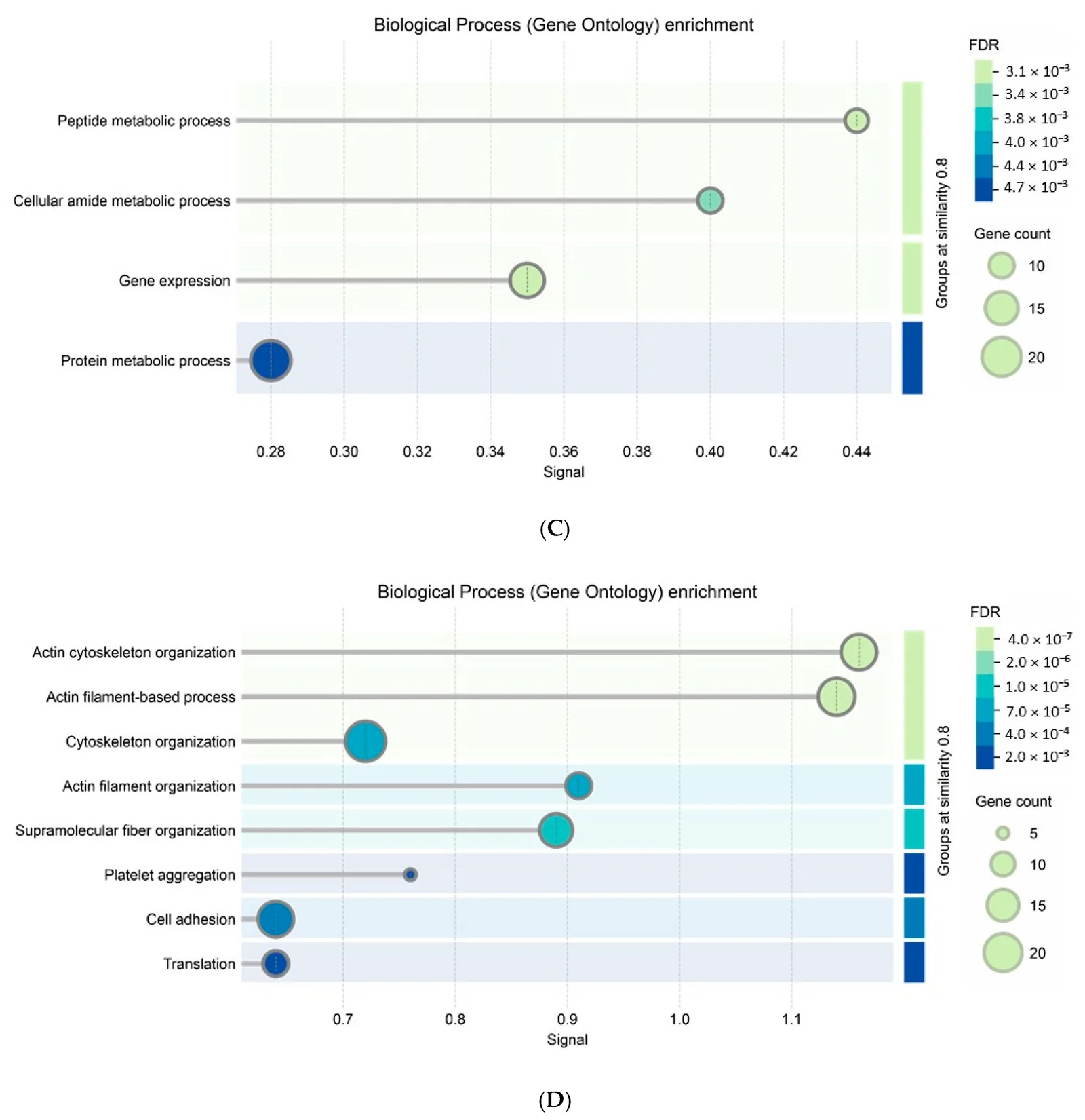
Plots showing pathway enrichment in significantly differing proteins (adjusted *p* < 0.05, fold change > 2) of biological processes according to the Gene Ontology Database. FDR is color-code and the number of proteins related to the pathway are plotted as circle sizes (legend for both is on the right). The X-axis represents Signal, a weighted metric of FDR and the observed/expected ratio, employed to avoid overemphasizing the more general terms: (**A**) proteins upregulated in the MSCs of patients at the onset of acute leukemia compared to donor MSCs; (**B**) proteins downregulated in the MSCs of patients at the onset of acute leukemia compared to donor MSCs; (**C**) proteins upregulated in the MSCs of patients on the remission of acute leukemia compared to donor MSCs; and (**D**) proteins downregulated in the MSCs of patients on the remission of acute leukemia compared to donor MSCs.

Pathway enrichment analysis using STRING-db identified a few significantly enriched pathways (Figure 3). Most of those pathways are linked to translation or cytoskeleton reorganization.

For further analysis, we manually selected the proteins related to cytoskeleton and supramolecular fiber organization that make up the group of extracellular matrix and adhesion proteins. We have, in addition, arbitrarily selected several protein groups related to MSC functions: mitochondrial proteins, proteins related to vesicular transport, and proteins related to differentiation. No significant changes were found in the abundance of proteins related to MSC differentiation. RRBP1 and GNB1 did not fit into any of these groups but warrant consideration. RRBP1 was downregulated both at the onset and in remission of leukemia. It is a core regulator of ribosome binding to endoplasmic reticulum and can drive cell survival in endoplasmic reticulum stress and inhibit apoptosis by activating TGF-β/SMAD PI3K/AKT and Notch pathways [23]. GNB1, on the contrary, was upregulated in both groups. It plays an important role in regulating transmembrane signal transduction [24].

### 2.2. Extracellular Matrix and Adhesion Proteins

ECM proteins were altered in the MSCs of patients with acute leukemia.

Many proteins were downregulated in the MSCs of patients both at the onset and in remission: COL6A2, THBS1, MYADM, CD44, PDLIM2, LMO7, P4HA1, BSG, COL1A2, RAP1B, COL5A1, PXN, FLNA, and FLNB. In addition, at the onset of acute leukemia, but not in remission, TRIOBP, LGALS1 and TGFBI were decreased. NID2 was upregulated in remission but not at the onset of the disease.

**Figure 4:**
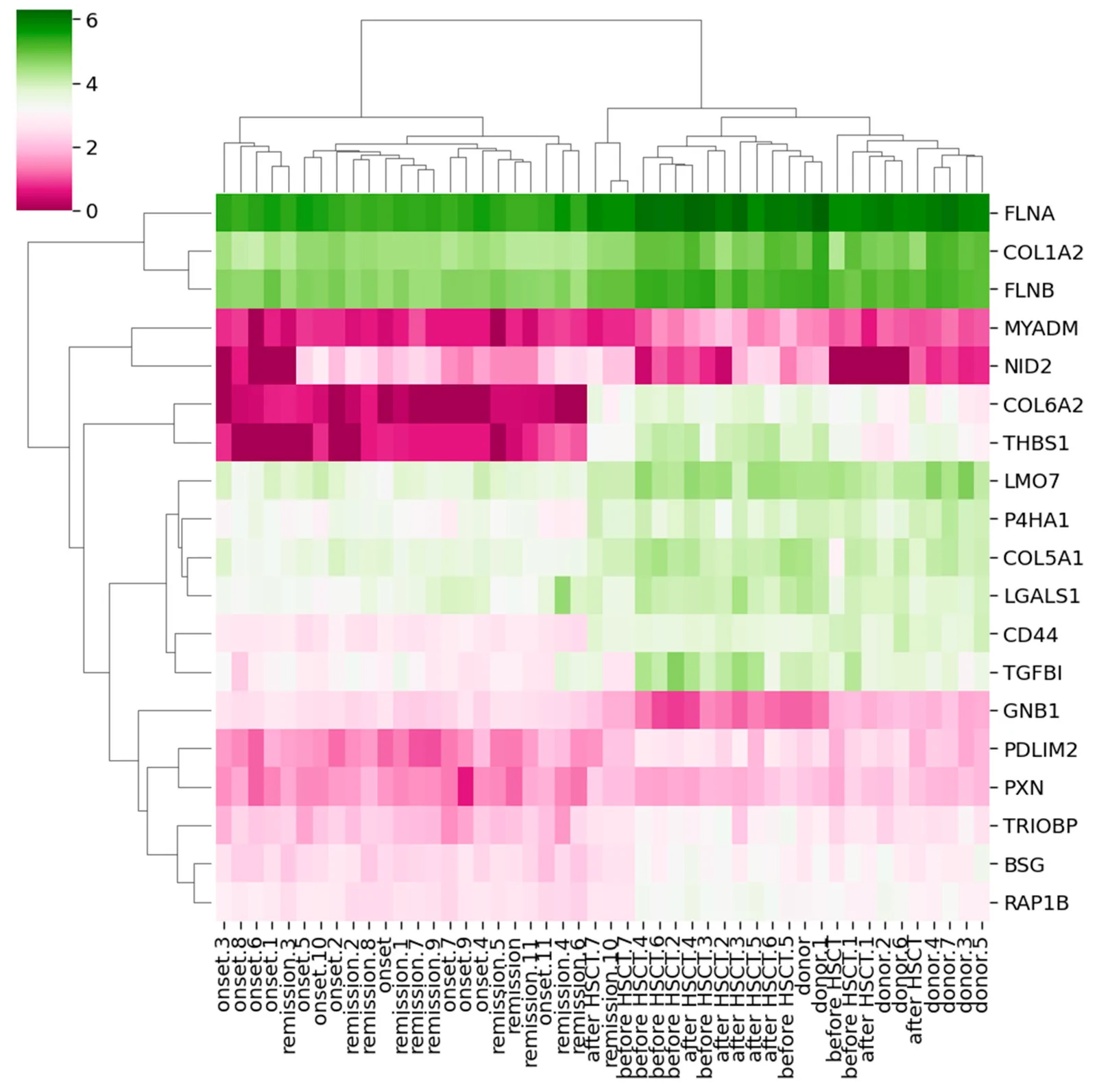
Clustering heatmap of differentially expressed proteins related to the extracellular matrix and adhesion.

The heatmap of differentially expressed proteins of extracellular matrix and adhesion is presented in Figure 4.

The clustering analysis shows that onset and remission expression patterns are similar, and that the donor cells’ pattern is closer to those of the MSCs of allo-HSCT patients.

### 2.3. Mitochondrial Proteins

At the onset of acute leukemia and in remission, MSCs’ proteomes differ from donors in the content of proteins related to mitochondria upkeep and activity, energy production, and antioxidant defense.

Many of the changes were observed in the MSCs of patients both at the onset and in remission: HSPE1-MOB4, MRPL21, and HSD17B4 were upregulated compared to donor MSCs, while CD44, COX2, DNM1L, ATP5F1B, ENO1, ALDOA, SLC16A3, PKM, APMAP, HSPE1, and OAT were all downregulated. In addition, at the onset of acute leukemia, but not in remission, NDUFS5 and CTBP2 were increased while PPP1CB, SLC25A6, and ERO1A were decreased in MSCs.

In remission, but not at the onset of the disease, MRPS31, PMPCA, CIAO1, TXNRD2, UBASH3B, ARMCX3, and PDPR were upregulated and NNMT, IMMT and GLUD1 were downregulated compared to donor cells.

**Figure 5:**
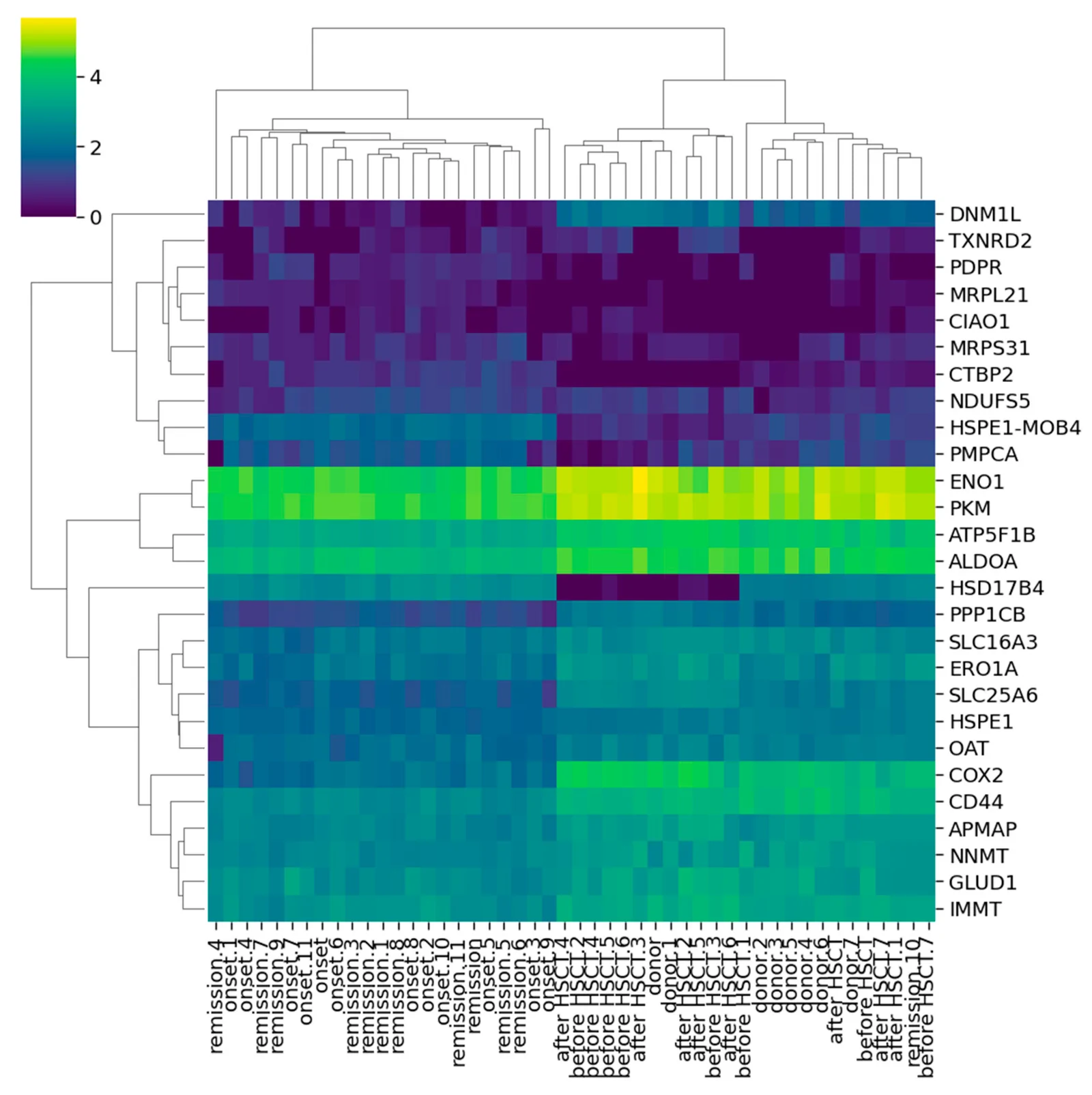
Clustering heatmap of differentially expressed mitochondrial proteins.

The heatmap of differentially expressed mitochondrial proteins is presented in Figure 5.

The clustering analysis shows that, as with the ECM proteins, onset and remission expression patterns are similar and that the donor cell patterns are closer to those of the MSCs of allo-HSCT patients.

### 2.4. Proteins Related to Vesicle Formation and Trafficking

As with the other groups, some of the changes observed in the MSCs of patients at the onset were also observed in remission. ANXA2, ANXA6, and VASP were downregulated in both groups. At the onset of acute leukemia, but not in remission, SGSH and EXOC6B were increased while PICALM and ARF4 were decreased compared to donors’ cells.

In remission, but not at the onset of the disease, TBC1D10A, HEATR5A, and UBASH3B were upregulated, and PLIN3 was downregulated.

**Figure 6:**
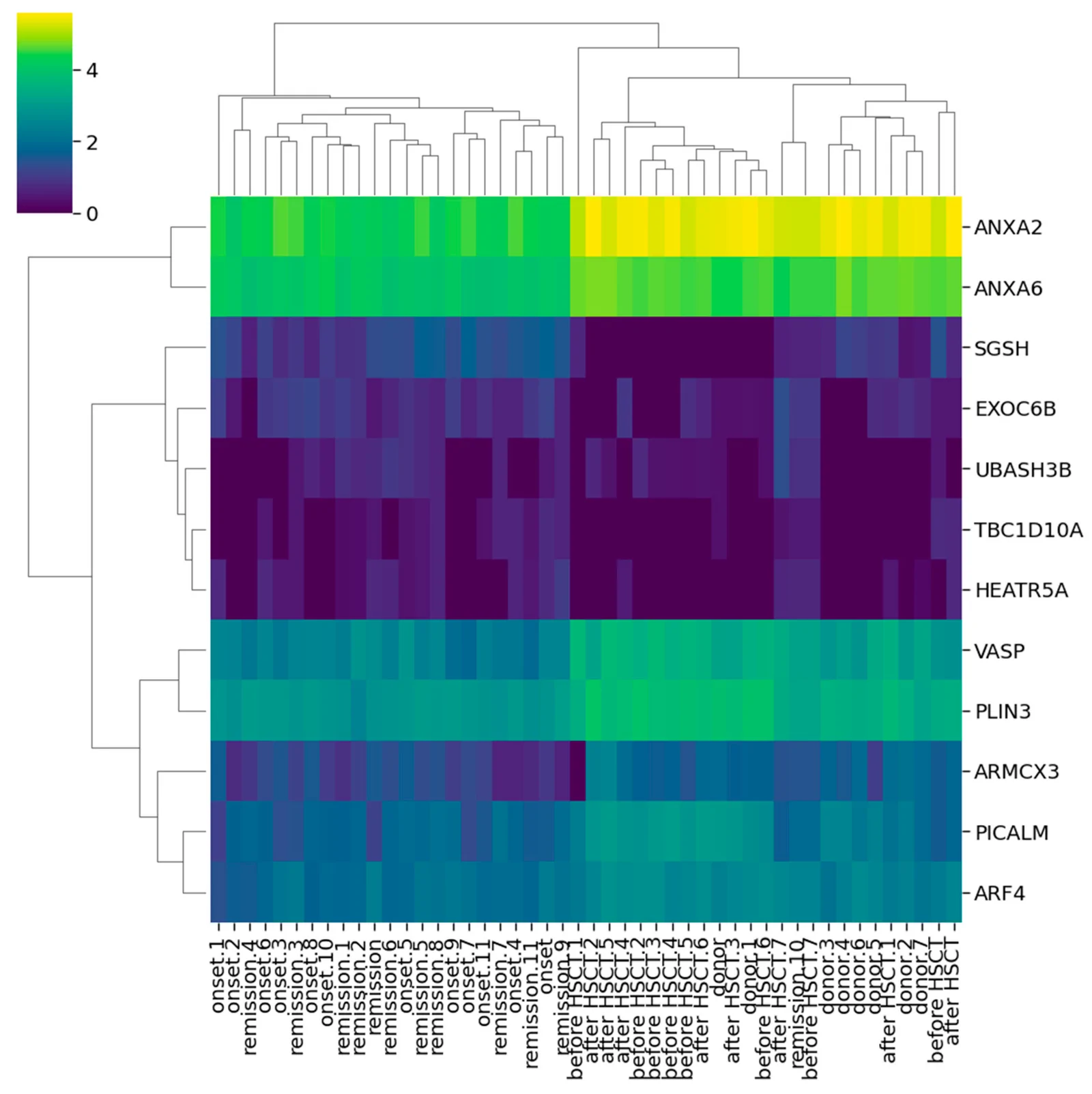
Clustering heatmap of differentially expressed proteins related to vesicular transport.

The heatmap of differentially expressed proteins related to vesicular transport is presented in Figure 6.

The clustering analysis shows that, like other groups of proteins, onset and remission expression patterns are similar, and donor cells’ pattern are closer to those of MSCs of allo-HSCT patients.

## 3. Discussion

### 3.1. General Proteome Discussion

The MSCs of acute leukemia patients exhibit poorer proliferation capacity than donor MSCs [25] and a portion of the patients’ bone marrow samples failed to yield MSC cultures feasible for LC–MS analysis. This impeded the obtainment of paired samples, which led to the sample size being a major limitation of this study. Another possible limitation is the uneven age and gender distribution between the donor and patient cohorts that stemmed from the same difficulties. In addition, ex vivo expansion, even over three passages, could select specific MSC subpopulations and modify the proteomic profile. As the donors’ cells had undergone the same cultivation procedures as the patients’ cells, we believe that the changes we detected in the comparisons are related to the disease/treatment rather than to cultivation.

The proteomes of acute leukemia patients’ MSCs differed from those of the donors at the onset and in remission. While the protein expression patterns were similar in samples obtained at the onset and in remission of the acute leukemia, a number of proteins differed from the donor level only at one of these points. The disappearance of some alterations and the emergence of others in remission can be attributed to either therapeutic agents or the elimination of leukemic cells; it was not possible to differentiate between the two influences within this study. STRING enrichment analysis revealed pathways affected differently in each group. The absence of a unified pattern of changes may be due to the small sample size in each group, the heterogeneity of patients in the groups, and other limitations of the study design. The biological processes that were seemingly affected both at the onset of acute leukemia and in remission are protein translation (GO terms such as translation, protein metabolic process, ribosome large subunit biogenesis) as well as supramolecular fibers and cytoskeleton formation, discussed as ECM and adhesion proteins (Section 2.2 and Section 3.2).

We were unable to identify any significant differences in the proteins of interest between the MSCs from patients before and after allo-HSCT and with the cells from healthy donors. Unexpectedly, donor samples differed most from the samples obtained at the onset and in remission of acute leukemia and were more similar to the samples before and after allo-HSCT. Our previous work, which explored growth characteristics and gene expression patterns, suggested the poor state of the MSCs of patients after allo-HSCT as a cause, supposedly because of the extensive pre-transplantation therapy regimen; the growth characteristics of the cells studied in this work display the same trend. However, we did not detect the same pronounced changes at the proteomic level. The minimum detectable effect size (Cohen’s d) with 80% power and a significance level of 0.05 was 1.507 for both the ‘before HSCT’ vs. ‘donors’ and ‘after HSCT’ vs. ‘donors’ comparisons. This is a very large effect size, which means that with the current sample size (eight samples in each group), we can only statistically detect very large differences between groups. Any true differences of a smaller magnitude are likely to remain undetected as statistically significant. This may explain the absence of significant differences in this study. Variability in the time points of sampling post HSCT, from 1 to 3 months, is another potential limitation factor. We understand that 1 month after allo-HSCT, the patient is only starting to recover hematopoiesis, while 2 months later, donor hematopoietic cells have already repopulated the recipient’s bone marrow. This inevitably affects the properties of MSCs. It is possible that the changes in bone marrow stroma are happening constantly and swiftly and level each other out when analyzed together. Nevertheless, we aimed to analyze paired samples and have accepted this limitation.

This is, to our knowledge, the first proteome study performed on the MSCs of acute leukemia patients, despite extensive research on leukemic cells proteomes and MSC features in leukemia. Lack of physiological validation limits the strength of our conclusions. Further functional studies will be able to clarify our findings.

### 3.2. Extracellular Matrix and Adhesion Proteins

ECM proteins contribute to regulation of cellular processes, including adhesion, signaling, differentiation, and intercellular communication.

Several ECM proteins decreased both at the onset and in remission of leukemia. Collagens COL6A2, COL1A2, COL5A1 are the key structural elements of ECM. P4HA1 is a collagen hydrolase required for collagen deposition and participation in ECM remodeling. P4HA1 is implicated as an oncogene in a variety of cancers and was suggested as a putative target for targeted therapy [26,27,28]. It is possible that it facilitates leukemia development as well, and, with additional insight into its role and effects, could be targeted. CD44 is a transmembrane protein involved in cell–cell interactions, cell adhesion, and migration. It is a receptor for hyaluronic acid and can also interact with other ligands such as osteopontin, collagens, and matrix metalloproteinases [29]. CD44 is present in the supramolecular complex containing BSG protein, which was decreased in both groups as well [30]. In line with our data, in myelodysplastic syndrome patients’ MSCs, the CD44 decrease correlated with a proliferative decline in cells [31]. It seems that the ECM structure formation by MSCs from acute leukemia patients may be disturbed.

Several proteins responsible for cell shape and adhesion were also downregulated in the MSCs of leukemia patients. MYADM is a transmembrane protein associated with ECM and is required for cell spreading and migration [32]. FLNA and FLNB regulate the mechanical properties and shape of cells by cross-linking actin filaments [33]. TRIOBP is also involved in the stabilization of the actin cytoskeleton and participates in adhesion junction formation [34]. LGALS1 is implicated in modulating cell–cell and cell–matrix interactions [35]. PDLIM2 links cytoskeleton to adhesion molecules and can inhibit β-catenin signaling [36]. PXN also contributes to the interactions of cells with ECM by supporting integrin clustering and cell adhesion [37]. Reductions in the RAP1B amount in MSCs also lead to decreased adhesion to ECM and, thus, to impaired cell migration [38]. LMO7 is required for migration along fibronectin fibers [39]. The downregulation of these proteins may be one of possible explanations for the abnormal adhesion of patients’ MSCs observed by others [40].

LMO7 also participates in the negative feedback regulation of TGF-β pathway [41]. TGF-β-induced protein (TGFBI) was decreased in MSCs at the onset of leukemia. TGFBI is an ECM component of particular importance for hematopoiesis, as it affects MSC interaction with hematopoietic precursors; it was shown that a decrease in TGFBI enhanced hematopoietic cell survival in the presence of MSCs [42].

Thrombospondin-1 (THBS1) is an ECM protein shown to act in the tumor microenvironment to inhibit angiogenesis, regulate antitumor immunity, stimulate tumor cell migration, and regulate the activities of extracellular proteases and growth factors [43]. A decrease in THBS1 observed in the proteomes of patients at the onset and in the remission of leukemia may be associated with aberrant angiogenesis and disruption of the normal signaling pathways in the bone marrow niche, further facilitating the leukemogenesis.

The only ECM protein upregulated in the MSCs from patients with acute leukemia, NID2, is increased exclusively in remission. It maintains the integrity and stability of the basement membrane by connecting laminin and collagen IV networks [44].

Therefore, we observed a major decrease in the production of proteins related to ECM formation, as well as MSC adhesion and migration, in the MSCs of acute leukemia patients. ECM and its proteins are vital to leukemogenesis; further orthogonal studies in the context of acute leukemia are needed.

### 3.3. Mitochondrial Proteins

The changes detected in mitochondrial protein composition may suggest a metabolic shift from glycolysis to oxidative phosphorylation both at the onset and in the remission of acute leukemia, which should be confirmed in future physiologic analyses.

A number of glycolysis-related proteins were decreased in patients’ cells compared to donors’, both at the onset of the disease and in remission: CD44, PKM2, ENO1, ALDOA, and SLC16A3. CD44 promotes pyruvate kinase M2 (PKM2) activity and, thus, enhances glycolysis. Its decrease may inhibit glycolysis and activate mitochondrial respiration, potentially leading to the depletion of reduced glutathione and the increase in ROS levels [45]. PKM2 is a key enzyme in aerobic glycolysis [46]. ENO1 catalyzes the inter-conversion of 2-phosphoglycerate to phosphoenolpyruvate [47]. ALDOA is a glycolytic enzyme, as well as a mitophagy and inflammation regulator [48]. SLC16A3 is a key regulator of lactic acid efflux [49].

These decreases align with the PDPR increase in remission. PDPR is an activating regulatory subunit of pyruvate–dehydrogenase phosphatase, a complex that activates pyruvate dehydrogenase and enhances acetyl-CoA synthesis, thus promoting mitochondrial respiration [50].

OAT, a pyridoxal 5′-phosphate-dependent enzyme, is a key contributor to glutamine supply [51]. It is also underrepresented in the proteomes of MSCs in patients both at the onset and in the remission of acute leukemia. GLUD1 is localized mostly in the mitochondria matrix and catalyzes reversible oxidative deamination of glutamate to α-ketoglutarate [52]. This enzyme is downregulated in MSCs in remission. The observed changes may hint at a possibility of metabolic imbalance in the stromal cells of acute leukemia patients.

Electron transport chain protein composition seems to be altered in acute leukemia. Among Complex I proteins, at the onset of the disease, MSCs contained more NDUFS5 compared to donor cells. In remission, NDUFS5 was still upregulated. On the other hand, Complex IV protein COX2 was decreased in the MSCs of patients both at the onset and in the remission of acute leukemia compared to those of donors, as was Complex V protein ATP5F1B. A imbalance between the proteins of the electron transport chain may possibly be associated with disruption of ATP synthesis, as has been described for Complex V in tumor cells [53,54]. CIAO1 catalyzes a step in Fe/S cluster biogenesis, with some of the said cluster being necessary later in the electron transport chain [55]. This protein was upregulated in remission. The observed changes suggest possible disruption of the respiratory chain in patients’ MSCs. This, in turn, may be associated with the metabolic stress induced by leukemia.

Changes in the proteins participating in transport through the mitochondrial membrane were also revealed in acute leukemia MSCs. A transmembrane translocase SLC25A6 imports ADP from cytosol into mitochondrion in exchange for ATP, supplying the cell with energy and fueling more ATP production. It was decreased in the proteomes of the patients at the onset of leukemia. It is a crucial link in the exchange of ADP and ATP through the mitochondrial inner membrane and is considered an anti-apoptotic agent [56]. PPP1CB, a catalytic subunit of protein phosphatase 1 that participates in metabolism and apoptosis regulation [57], decreased at the onset of leukemia as well.

Fatty acid β-oxidation is very important for mitochondrial energy production. HSD17B4, a β-oxidation enzyme [58], was overexpressed in the MSCs of patients at the onset and in remission of acute leukemia.

ERO1α is part of the key molecular machinery that enables fast metabolic adaptation to stress and enhances mitochondrial oxidative phosphorylation [59]. Its downregulation at the onset of leukemia may reflect the impaired stress-responsive abilities of MSCs. NNMT is a metabolic enzyme also involved in stress-associated metabolic and epigenetic regulation [60]. It was found to be reduced in remission only, suggesting that the stress response of stromal cells may possibly be weakened at that moment.

The proteins regulating the mitochondrial structure were affected in the MSCs of acute leukemia patients. IMMT is a key regulatory subunit of mitochondrial integrity. It is an important part of the mitochondrial contact sites and cristae organization system complex [61]. Its reduction in remission hints to structural defects in mitochondria. HSPE1-MOB4 functions as a co-chaperonin in mitochondrial protein folding. It was increased in the MSCs of patients at the onset and in the remission of acute leukemia. On the contrary, HSPE1, which is also a co-chaperone that resides in the mitochondrial matrix [62], was decreased in those groups. Depletion of HSPE1 can lead to mitochondria fission and a decrease in fusion. On the other hand, DNM1L was decreased both at the onset and in remission. DNM1L is required for mitochondrial fission [63]; therefore, its decrease can potentially contribute to a shift toward fusion. A combination of the described changes may suggest a disrupted mitochondrial structure.

A part of mitochondrial proteins, including the majority of electron transport chain proteins, is synthesized by mitochondrial ribosomes as opposed to cytosolic ribosomes. MRPs that make up the mitochondrial ribosomes are essential for mitochondrial maintenance and function. In the MSCs of patients at the onset and in remission of acute leukemia, MRPs and some of the other proteins involved in translation were dysregulated. MRPL21 upregulation, detected in both groups, may inhibit apoptosis, potentially through modulation of the P53 pathway [64]. MRPS31 is upregulated in MSCs in remission only. Studies suggest the existence of a negative feedback loop between MRSPS31 and COL1A1 through the TFB1 pathway [65]. This may explain the downregulation of collagens described above. UBASH3B specifically dephosphorylates MRPL12, downregulating mitochondrial metabolism [66]. The upregulation of UBASH3B in remission may be involved in a shift in cells’ respiration towards oxidative phosphorylation. PMPCA is a subunit of the mitochondrial processing peptidase complex that allows for mitochondrial protein maturation by cleaving precursors [67]. This protein is also increased in remission.

In remission, a potent antioxidant TXNRD2 [68] is increased. ABMAP is increased both at the onset and in remission; it protects cells from ferroptosis—a variant of programmed cell death triggered by excessive accumulation of lipid hydroperoxides and ROS [69]. ARMCX3 has been proven to be tightly associated with mitochondrial dynamics through positive regulation of ROS production [70]. It is downregulated in MSCs in remission, which may lead to the possible inhibition of ROS production. It seems that antioxidant regulation may be activated in the stroma at the onset and in the remission of acute leukemia.

CTBP2, an important metabolite sensor, is secreted via exosomes in response to reductive metabolism, which is suppressed by oxidative stress [71]. It is upregulated in MSCs at the onset of leukemia, once again reflecting possible compensatory mechanisms.

### 3.4. Proteins Related to Vesicle Formation and Trafficking

In acute leukemia patients’ MSCs, levels of vesicular transport and membrane trafficking proteins are also altered compared to donor cells. Potentially, this may be associated with possible disbalance in intercellular communication via exosomes and vesicles.

ANXA2 and ANXA6 enable membrane lipid segregation, membrane deformation, and vesiculation [72,73]. They are decreased both at the onset and in the remission of acute leukemia. VASP is a key actin-regulatory protein mediating phagosome formation [74]. It is also decreased both at the onset and in the remission of acute leukemia. PICALM, decreased at the onset of acute leukemia, is involved in autophagy [75,76]. This protein binds to clathrin and is important for vesicle formation. ARF4 is involved in transport between the endoplasmic reticulum and the Golgi and plays a role in tubule formation [77]. This protein is also downregulated in MSCs at the onset of leukemia. All these changes may suggest possible impairment of phagocytosis and vesiculation in patients’ MSCs. This suggestion should definitely be proven through direct experiments.

In remission, PLIN3 is underrepresented. This protein is involved in lipid droplets formation and intracellular vesicular trafficking [78]. The observed decrease may possibly affect MSCs differentiation to adipocytes as well as vesicular formation.

At the onset, EXOC6B and SGSH are overexpressed in MSCs. EXOC6B is necessary for exocytosis [79]; thus, it is crucial for cell growth and migration. SGSH is an essential enzyme located within lysosomes and is specific for heparin sulfate degradation [80].

In remission, TBC1D10A and HEATR5A are upregulated. TBC1D10A negatively regulates cilium length, function, and membrane composition [81]. It inhibits protein recruitment to damaged mitochondria and to autophagosomes, thereby postponing mitophagy and the maturation of autophagosomes [82]. The HEATR5A protein localizes to the trans-Golgi network and endosomes and participates in membrane traffic [83].

Analysis of the proteomes of MSCs from the bone marrow of acute leukemia patients revealed alterations in proteins associated with ECM regulation, vesicular trafficking, and mitochondrial proteins. Some changes detected in MSCs at the onset of the disease have also been detected in remission.

## 4. Materials and Methods

### 4.1. Patients

The MSC proteome analysis included bone marrow MSC samples from 12 acute leukemia patients (4 ALL, 8 AML) at the onset and the same patients in remission; and 8 patients (4 ALL, 4 AML) before, and 1–3 months after, allo-HSCT. Clinical data are presented in the Supplement Materials (Tables S3 and S4). MSCs from 8 healthy donors were used as a control (Table 2). Bone marrow samples from healthy donors were obtained during bone marrow donation for transplantation; bone marrow samples from patients with acute leukemia were obtained during diagnostic punctures. All work was conducted in accordance with the Declaration of Helsinki (1964). This study was approved by the local ethics committee; donors and patients provided written informed consent.

### 4.2. MSCs Cultivation

The bone marrow was separated on 0.1% methylcellulose (1500 cp, Sigma-Aldrich, St. Louis, MO, USA). Mononuclear cells were seeded at 3 × 10^6^ in T25 culture flasks (Corning-Costar, Corning, NY, USA) in a standard culture medium consisting of alpha-MEM supplemented with 10% fetal bovine serum (HyClone, Logan, UT, USA), 2 mM L-glutamine (ICN, Freehold, NJ, USA), 100 U/mL penicillin (Sintez, Moscow, Russia), and 50 μg/mL streptomycin (BioPharmGarant, Vladimir, Russia). Cultures were carried out at 37 °C and 5% CO_2_.

After reaching confluence, the cells were washed with 0.02% EDTA (ICN, Freehold, NJ, USA) in 0.9% NaCl (Sigma-Aldrich, St. Louis, MO, USA) and then detached by treatment with 0.25% trypsin (ICN, Freehold, NJ, USA) (passage 0). The number of collected cells was counted directly; cell viability was determined by Trypan blue dye exclusion. During passage, cells were plated at a concentration of 4 × 10^3^ cells per cm^2^ of flask bottom.

The cells fit the MSC criteria: spindle-shaped morphology, plastic adherence, and standard surface phenotype (CD105+ CD73+ CD90+ CD45− CD34− CD33− CD117− CD13−). We analyzed the phenotype of MSCs on passage 1 and the differentiation abilities on passage 2. The osteogenic differentiation medium was supplemented with 0.1 µM dexamethasone (Sigma-Aldrich, St. Louis, MO, USA), 0.15 mM ascorbic acid 2-phosphate trisodium salt (ICN, Freehold, NJ, USA), and 3 mM NaH_2_PO_4_ (ICN, Freehold, NJ, USA). The adipogenic differentiation medium was supplemented with 1 µM dexamethasone (Sigma-Aldrich, St. Louis, MO, USA), 60 µM indomethacin (Sigma-Aldrich, St. Louis, MO, USA), and 5 µg/mL insulin (Sigma-Aldrich, St. Louis, MO, USA). Successful differentiation was confirmed by either gene expression analysis (*FABP4* and *PPARG* for adipogenic differentiation and *ALPL*, *PTHR1*, *BGLAP* and *SPP1* for osteogenic differentiation) or specific staining (Oil Red O for adipogenic differentiation and Alizarin Red for osteogenic). Phenotypic and differentiation characterization were performed only for representative cultures (no less than a half in each group).

The proteome was analyzed at passage 3 in order to obtain the cell numbers necessary for analysis and avoid both the admixture of hematopoietic lineage cells that can be retained in earlier passages as well as the genetic aberrations that can occur in later passages.

### 4.3. Preparation of MSCs Samples for Proteome Analysis

The cells were washed with PBS, incubated in serum and phenol red free media for 24 h, and then detached from the flask bottom with a cell scraper and centrifuged for 10 min at 450 *g*; the resulting dry pellet was stored at −70 °C.

### 4.4. Mass Spectrometry Sample Preparation

A protease inhibitor cocktail (Halt Protease Inhibitor Cocktail, Thermo Scientific, Waltham, MA, USA) was added to each sample; the samples were then centrifuged at 1500 *g* for 10 min to remove debris. Supernatants were immediately frozen and lyophilized to reduce volume. The lyophilized samples were resuspended for 30 min in a buffer containing 6 M Gd–HCl, 10 mM TRIS–HCl (pH 8) and 2 mM DTT. To precipitate the insoluble fraction, the solutions were centrifuged at 16,000 *g* for 10 min at 4 °C. Samples were concentrated using a centrifuge filter (Corning Spin-X UF6, Sigma-Aldrich, St. Louis, MO, USA) to replace the buffer. Buffer (8 M urea, 2 M thiourea, 10 mM TRIS–HCl (pH = 8)) was added to the concentrated samples at a ratio of 1:3 and incubated at room temperature for 30 min. Disulfide bonds were reduced with 5 mM DTT at room temperature for 40 min and then alkylated with 10 mM iodoacetamide in the dark, at room temperature, for 20 min. Alkylated samples were diluted by adding 50 mM NH4HCO3 solution at a ratio of 1:4; then, trypsin was added (0.01 μg per 1 μg of protein) and the samples were incubated at 37 °C for 14 h. The reaction was stopped by adding formic acid to a final concentration of 5%. The peptides were desalted using Discovery DSC-18 (tubes 1 mL, 50 mg) (Sigma-Aldrich, St. Louis, MO, USA), dried in a vacuum, and stored at −80 °C before analysis. Prior to LC–MS/MS, samples were redissolved in 5% acetonitrile with 0.1% trifluoroacetic acid and sonicated.

### 4.5. LC–MS/MS Analysis

Proteomic analysis was performed on an Orbitrap Q Exactive HF-X (Thermo Fisher Scientific, Waltham, MA, USA) mass spectrometer equipped with a nano-electrospray (nano-ESI) source and a high-pressure nanoflow chromatograph UPLC Ultimate 3000 (Thermo Fisher) equipped with a lab-packed reverse-phase (Kinetex C18, 2.4 μm, Phenomenex Inc., Torrance, CA, USA) column (100 mkm × 500 mm). The temperature of the column was thermostatically controlled at 60 °C. Samples were loaded in buffer A (0.1% Formic acid) and eluted with a linear (180 min) gradient of 3 to 55% buffer B (0.1% Formic acid, 80% Acetonitrile) at a flow rate of 220 nL/min. Mass spectrometric data were stored during automatic switching between MS1 scans and up to 16 MS/MS scans (topN method). The target value for MS1 scanning was set to 3 × 106 in the range 390–1400 *m*/*z* with a maximum ion injection time of 45 ms and a resolution of 60,000. The precursor ions were isolated at a window width of 1.4 *m*/*z*. Precursor ions were fragmented by high-energy dissociation in a C-trap with a normalized collision energy of 30 eV. MS/MS scans were saved at a resolution of 15,000 at 400 *m*/*z* and at a value of 2∙105 for target ions, with a maximum ion injection time of 50 ms.

### 4.6. Protein Identification and Bioinformatics Analysis

Raw LC–MS/MS data from Q Exactive HF mass-spectrometer were converted to *.mgf peaklists with MSConvert (release 3.0.20287, ProteoWizard Software Foundation, Palo Alto, CA, USA). For this procedure, we used the following parameters: “--mgf --filter peakPicking true [1,2]”. For thorough protein identification, the generated peak lists were searched with MASCOT (version 2.5.1, Matrix Science Ltd., London, UK) and X! Tandem (ALANINE, 2017.02.01, The Global Proteome Machine Organization, New York, NY, USA) search engines against UniProt Human Protein Knowledgebase with the concatenated reverse decoy dataset. The precursor and fragment mass tolerance were set at 20 ppm and 0.04 Da, respectively. Database searching parameters included the following: tryptic digestion with one possible missed cleavage, static modification for carbamidomethyl (C), and dynamic/flexible modifications for oxidation (M). For X!Tandem, parameters were chosen that allowed us to quickly check the acetylation of the N-terminal residue of the protein, the loss of ammonia from the N-terminal glutamine and the loss of water from the N-terminal glutamic acid. The results from the search engines were integrated in Scaffold 5 (version 5.1.0). An algorithm for estimating the local false discovery rate (FDR) with the standard grouping of proteins was used. To assess the hits of peptides and proteins, FDR = 0.05 was chosen for both. The samples annotated in the Swiss-Prot database were marked as preferred.

### 4.7. Statistical Analysis

A semi-quantitative approach based on spectral counting (PSMs, peptide-to-spectrum matches) was used to quantify proteins. Missing values were not imputed. Data were normalized by the total PSMs. Proteins were considered to be differentially downregulated if their log 2-fold change was less than −1, and the *p*-value was less than 0.05 (after FDR correction). Proteins were considered to be differentially upregulated if their log 2-fold change was more than 1, and the *p*-value was less than 0.05 (after FDR correction).

Statistical analysis was performed using GraphPad Prism, version 8.03 (GraphPad Software Inc., Boston, MA, USA). Due to the non-normal distribution of the data, the Mann–Whitney U-test was used for comparison of non-paired samples and the Wilcoxon matched-pairs signed-rank test was used for paired samples. Benjamini–Hochberg multiple testing (FDR) correction was applied to all data.

Volcano plots, PCA and heatmaps were created in Python 3, using the Pandas 3.0.4, Matplotlib 3.11.0 and Seaborn libraries 0.13.2. The mean abundance of each protein was log transformed (applied to all data) for normalization in each group. The hierarchical clustering was conducted using Seaborn’s clustermap() function.

For pathway enrichment analysis, lists of differentially secreted proteins were uploaded onto the STRING-db online service. The service uses Benjamini–Hochberg multiple testing correction for FDR; the Signal metric was used to avoid favoring general terms. Signal was computed as a weighted harmonic mean between the observed/expected ratio and −log(FDR). The pathways were grouped by a gene ontology similarity ≥ 0.8.

## 5. Conclusions

Studies of the MSC proteome in patients with acute leukemia have shown that the proteins responsible for extracellular matrix formation and function, cell adhesion, vesicular transport, intercellular interactions, and mitochondrial activity were significantly altered. Unexpectedly, our study did not reveal any differences between the proteomes of MSCs from patients before and after allo-HSCT. The proteomes did not differ from the proteomes of healthy donors either.

Considering the onset changes observed in the proteins involved in ECM formation, mitochondrial function, and vesicular trafficking, an increased demand for the stromal microenvironment to supply leukemic growth could be implied. Most parts of these processes continue in remission.

## Figures and Tables

**Table 1:** Growth characteristics of cultures of MSCs from the bone marrow of patients with acute leukemia and healthy donors. Data are given as the median [Quartile 25–Quartile 75].

Comparison Group	MSCs Cumulative Production, ×10^6^ Cells	Time to P0, Days	Time to P3, Days	Population Doubling Time, Days
Donors (N = 8)	11.0 [10.4–21.8]	10.5 [10–12]	24.5 [24–26]	2.0 [1.7–2.9]
Onset (N = 12)	8.5 [3.8–13.3]	15 [13–17] *	34 [28–37] *	3.4 [2.1–4.7]
Remission (N = 12)	12.4 [10.3–16.9]	14 [13–15]	30 [28–31]	2.3 [2.0–2.8]
Before allo-HSCT (N = 8)	12.0 [8.8–17.2]	13 [12–14]	28 [27–30]	2.4 [1.9–2.9]
After allo-HSCT (N = 8)	8.0 [6.3–9.3] **	17 [14–20] *,**	34 [28–39] *	3 [2.6–3.6]

* indicates *p* < 0.05 when compared to donors; ** indicates *p* < 0.05 when compared to before allo-HSCT.

**Table 2:** Characteristics of patients and donors.

	N	Age, Years (Median)	Gender, Male/Female
Donors	8	14–41 (30.5)	7/1
Onset and Remission	12	19–56 (33.5)	4/8
Before and After allo-HSCT	8	22–56 (38)	3/5

## Data Availability

The data presented in this study are available from the corresponding author on reasonable request.

## Supplementary Materials

The following supporting information can be downloaded at: https://www.mdpi.com/article/10.3390/ijms27167402/s1.
